# Colorectal adenomas and diet: a case-control study of subjects participating in the Nottingham faecal occult blood screening programme.

**DOI:** 10.1038/bjc.1993.31

**Published:** 1993-01

**Authors:** J. Little, R. F. Logan, P. G. Hawtin, J. D. Hardcastle, I. D. Turner

**Affiliations:** SEARCH Programme, Unit of Analytical Epidemiology, International Agency for Research on Cancer, Lyon, France.

## Abstract

Diets high in animal fat and protein and low in fibre and calcium are thought to be factors in the etiology of colorectal cancer. Intakes of these nutrients were determined in three groups participating in a randomised trial of faecal occult blood (FOB) screening. A diet history was obtained by interview from 147 patients with colorectal adenomas, 153 age and sex matched FOB-negative controls (a) and 176 FOB-positive controls without colorectal neoplasia (b). Unconditional logistic regression was used to estimate relative risks (RR) and 95% confidence limits (increases) adjusted for age, sex and social class. After adjustment for total energy intake, no associations were found with total, saturated or mono-unsaturated fat, or calcium intake. For total fibre intake there were non-linear relationships with both control groups with the crude RR for highest quintiles of total fibre intake compared to the lowest being 0.6, although this pattern was no longer apparent after adjustment for energy intake with group (a). In comparison with group (b) cereal fibre intake showed a more consistent inverse relationship with adenoma prevalence with the RR for ascending quintiles of intake being 1.0, 0.7 (0.3-1.6), 0.5 (0.3-1.1), 0.7 (0.4-1.4) and 0.3 (0.1-0.6) (trend chi 2 = 8.80, p = 0.003). In comparison with group (a), the adjusted RR for the highest quintile of cereal fibre intake compared with the lowest was 0.6, but no clear trend was apparent. There was an unexpected positive relationship between adenomas and polyunsaturated fat intake with the RR for having an adenoma being 1.0, 2.8 (1.3-6.1), 1.6 (0.7-3.4), 3.5 (1.6-7.5) and 2.3 (1.1-5.0) for ascending quintiles of polyunsaturated fat intakes (trend chi 2 = 4.8, P = 0.03) in comparison with group (a) only. Our data, while providing no support for the role of dietary animal fat or protein, do support the protective role of dietary cereal fibre in the etiology of colorectal adenomas.


Br. J. Cancer (1993), 67, 177–184 © Macmillan Press Ltd., 1993

### Colorectal adenomas and diet: a case-control study of subjects participating in the Nottingham faecal occult blood screening programme

J. Little[1,2], R.F.A. Logan[2], P.G. Hawtin[2], J.D. Hardcastle[3] & I.D. Turner[2]

[1]*SEARCH Programme, Unit of Analytical Epidemiology, International Agency for Research on Cancer, 150 cours Albert-Thomas, F-69372-Lyon Cedex 08, France;* [2]*Department of Public Health Medicine and Epidemiology, University of Nottingham Medical School, Queen's Medical Centre, GB-Nottingham NG7 2UH;* [3]*Department of Surgery, University of Nottingham Medical School, Queen's Medical Centre, GB-Nottingham NG7 2UH, UK.*

**Summary** Diets high in animal fat and protein and low in fibre and calcium are thought to be factors in the etiology of colorectal cancer. Intakes of these nutrients were determined in three groups participating in a randomised trial of faecal occult blood (FOB) screening. A diet history was obtained by interview from 147 patients with colorectal adenomas, 153 age and sex matched FOB-negative controls (a) and 176 FOB-positive controls without colorectal neoplasia (b). Unconditional logistic regression was used to estimate relative risks (RR) and 95% confidence limits (↑) adjusted for age, sex and social class.

After adjustment for total energy intake, no associations were found with total, saturated or mono-unsaturated fat, or calcium intake. For total fibre intake there were non-linear relationships with both control groups with the crude RR for highest quintiles of total fibre intake compared to the lowest being 0.6, although this pattern was no longer apparent after adjustment for energy intake with group (a). In comparison with group (b) cereal fibre intake showed a more consistent inverse relationship with adenoma prevalence with the RR for ascending quintiles of intake being 1.0, 0.7 (0.3–1.6), 0.5 (0.3–1.1), 0.7 (0.4–1.4) and 0.3 (0.1–0.6) (trend $\chi^2 = 8.80$, p = 0.003). In comparison with group (a), the adjusted RR for the highest quintile of cereal fibre intake compared with the lowest was 0.6, but no clear trend was apparent. There was an unexpected positive relationship between adenomas and polyunsaturated fat intake with the RR for having an adenoma being 1.0, 2.8 (1.3–6.1), 1.6 (0.7–3.4), 3.5 (1.6–7.5) and 2.3 (1.1–5.0) for ascending quintiles of poly-unsaturated fat intakes (trend $\chi^2 = 4.8$, $P = 0.03$) in comparison with group (a) only.

Our data, while providing no support for the role of dietary animal fat or protein, do support the protective role of dietary cereal fibre in the etiology of colorectal adenomas.

High intakes of animal fat and protein (Wynder & Shigematsu, 1967; Drasar and Irving, 1973), and low intakes of fibre (Burkitt, 1971) and calcium (Newmark *et al.*, 1984) have been postulated to increase the risk of colorectal cancer. However, the role of these nutrients has not been clarified in analytical epidemiological studies (Zaridze, 1983; Willett, 1989*a*). As it is generally accepted that colorectal carcinomas develop from adenomatous polyps, studies of subjects with these precursor lesions should lead to the identification of factors involved in the development of colorectal cancer. We have therefore investigated the relationship between diet, in particular dietary fat, protein and fibre and asymptomatic colorectal adenomas in subjects identified in a trial of faecal occult blood screening.

### Material and methods

### *Recruitment of subjects*

Subjects were recruited from amongst those performing faecal occult blood (FOB) tests administered in a trial of screening for colorectal cancer in Nottingham, described elsewhere (Hardcastle *et al.*, 1989).

Cases were subjects found to have adenomatous colorectal polyps following a positive FOB test. Only cases with histologically confirmed adenomas were included.

Two types of control were recruited for each case. First, subjects found to be FOB negative; participation was sought from the next subject in the screening trial records who was FOB negative and matched with the adenoma patient on age and sex. As the screening trial was carried out on a practice by practice basis, cases and controls were effectively matched on general practice. Second, patients who were FOB positive on screening but found to be free of adenomas and carcinomas on examination by colonoscopy or barium enema.

FOB positive subjects and their controls were invited to participate in the present study once any hospital investigation and treatment as a consequence of screening had been completed. The screening trial from which subjects in the present study were recruited started in 1981, and we included subjects who had completed FOB tests up to 30 June 1988. We interviewed subjects between November 1985 and September 1988.

### *Data collection*

Information on dietary habits, height and weight, occupational history, leisure activity, demographic factors and medical history was obtained by an interview conducted at the subject's home by specially trained interviewers.

To facilitate the subject's recall, the methods of recording varied according to the type of food. The family's weekly consumption of fats was recorded at the interview along with the number of adults and children in the family. Respondents were asked to think in terms of what they ate in a typical week during the year prior to test notification. They were asked to describe what they would have for breakfast, what they would have for their main meals and their snacks and so forth. Further details of the diet history method are given in Jackson *et al.* (1990). To convert the dietary information into estimated nutrient intakes, the basic calculation was weight per week times concentration as estimated from the computerised McCance and Widdowson tables (Paul & Southgate, 1978).

In addition to the nutrients about which we had specific hypotheses, we considered total energy intake as an important potential confounder (Willett, 1989*b*, pp 245–271), the sources of protein and fibre, and the different types of fatty acid.

We also asked subjects about the frequency of consumption of certain foods which might be markers of 'healthy

Correspondence: J. Little, International Agency for Research on Cancer, 150 cours Albert-Thomas, F-69372 Lyon Cedex 08, France.
Received 29 November 1991; and in revised form 3 August 1992.

eating' as exemplified by the National Advisory Committee on Nutritional Education report (1983). These were chicken and fish as markers of lean meat consumption, yoghurt and fruit as markers of 'high fibre' diets and beef, cheese and biscuits as markers of a diet high in fats. As possible markers of fat intake, we also asked about grilled or fried foods preference, fat consumption compared with that of the spouse, fat eaten on different meats, consumption of chips and the use of fat in cooking them.

*Repeatability*

Agreement in tertile ranking between the diet history interview used and a validated questionnaire completed either 4 to 6 weeks before or four to six weeks after the diet history interview was found to be 58% for fibre, 53% for fat and 49% for calcium (Jackson *et al.*, 1990). In addition, 34 subjects (16 men) had repeat interviews in the same periods in each year and by the same interviewer. The correlation between reported intakes of fat was 0.58, of protein 0.47, and of fibre 0.81 (Table I). Adjusting for any correlation between nutrient intakes and overall reported intake had little effect except for fat. Correlations between reported intakes of most of the other nutrients were of the order of 0.6–0.8. Poor agreement ($r = 0.23$) was observed for cruciferous vegetables and for cholesterol ($r = 0.25$) after adjustment for total energy intake. Seven subjects reported a change in intake between interviews; three a change in fibre intake and four a change in fat intake. The overall measures of agreement were little changed when these subjects were excluded.

*Analysis*

For the analyses involving specific nutrients, quintiles for each factor were formed for the total number of subjects in each set of comparisons (Hsieh *et al.*, 1991).

The relative risk estimates (RR) are odds ratios as calculated by the Mantel-Haenszel technique using the SEARCH package (Macfarlane *et al.*, 1991) and by unconditional logistic regression, using the GLIM package (Baker *et al.*, 1985) with routines developed by Maisonneuve *et al.* (paper submitted). Adjustment for age, sex and socio-economic status was made in all analyses, thus obviating the need for a matched analysis (Rothman, 1986), and the consequent loss of data from incomplete matched pairs. However, a matched analysis was also performed using the 129 matched pairs available for the main hypotheses relating to dietary fat and fibre intakes. In the comparison with FOB positive subjects, adjustment was made also for interactions between age and sex, age and socio-economic status, as this improved the fit of the 'core' model.

Other variables considered as potential confounders included physical activity, body size, aspects of medical history and history in first-degree relatives of large bowel cancer, breast cancer, other cancer, heart disease or stroke, year of notification, year of interview and the interval between these. In analyses relating to fat and protein, adjustment for total energy intake was made by the nutrient residuals technique (Willett & Stampfer, 1986). The chi-square test for trend was applied where appropriate. The goodness-of-fit of the logistic regression models was assessed by the test described by Hosmer and Lemeshow (1989). An adequate fit was obtained for all but one of the models (see Table IV) reported in this paper. We also investigated associations with adenomas defined by size, histological type (tubular only or with villous elements) and multiplicity. As FOB positive controls with inflammatory bowel disease, diverticular disease or coeliac disease might have changed their diet, we repeated the analyses based on the FOB positive group with these subjects excluded.

**Table I** Correlation between nutrient intakes reported in original and repeat interviews, based on 34 subjects

| | Correlation coefficients | |
|---|---|---|
| *Nutrient* | *Crude* | *Adjusted for energy intake* |
| Energy | 0.57 | – |
| Fat | 0.58 | 0.33 |
| Saturated | 0.46 | 0.38 |
| Monounsaturated | 0.45 | 0.32 |
| Polyunsaturated | 0.62 | 0.63 |
| Protein | 0.47 | 0.49 |
| Animal | 0.52 | 0.46 |
| Meat | 0.38 | 0.31 |
| Vegetable | 0.60 | 0.68 |
| Cholesterol | 0.45 | 0.25 |
| Fibre | 0.81 | 0.85 |
| Cereal fibre | 0.84 | 0.85 |
| Cruciferous vegetables | 0.23 | 0.24 |
| Calcium | 0.83 | 0.78 |
| Phosphorous | 0.72 | 0.87 |
| Vitamin D | 0.67 | 0.53 |
| Other nutrients | | |
| Carbohydrate | 0.83 | 0.67 |
| Retinol | 0.49 | 0.48 |
| Carotene | 0.61 | 0.58 |
| Vitamin C | 0.59 | 0.58 |
| Vitamin E | 0.69 | 0.70 |
| Iron | 0.62 | 0.59 |

### Results

*Composition of the study groups*

Between January 1981 and June 1988, 606 trial subjects were found to be FOB positive. In 222 a polyp, though to be adenomatous, was found. Of these, 29 could not be approached because they had either died, moved away or were regarded as unfit to be interviewed. Of the remaining 193, 169 (88%) were interviewed but in 22 the polyp was either not retrieved (9) or not adenomatous on histological examination (13).

Of the other 384 FOB positive subjects, 68 had cancers, 62 were randomly excluded for logistic reasons from two practices which had used an FOB test giving a high false positive rate (Armitage *et al.*, 1985) and 37 could not be approached for reasons similar to the cases. Of the remaining 217, 176 (81%) were interviewed.

Of the FOB negative subjects initially identified 41 could not be approached and were replaced because they had either died (7), moved away (11) or were regarded as unfit to be interviewed (23). Of the 169 eventually approached 153 (91%) were interviewed.

The socio-demographic characteristics of the groups are summarised in Table II. Although not statistically significant, the distributions according to the socio-economic status associated with the job held for the longest period differed between cases and FOB negative controls. When adjustment was made for this measure, no associations with other measures of socio-economic status based on the occupational data were found. There were no notable differences between the groups in mean school leaving age, level of education since leaving school, marital status, length of residence in the Nottingham area or place or birth.

*Size, histological type and multiplicity of adenomas*

Of the 122 subjects in whom the site of the adenoma was recorded, in 96% the adenomas were located in the descending colon, rectosigmoid or rectum. The adenomas were recorded as being less than 1 cm in maximum diameter or 'small' in 42 cases, as 1–1.9 cm or 'medium' in 70 cases, and as 2 cm or more in maximum diameter or 'large' in 30 cases. For the 34 cases with more than one adenoma, size was categorised according to the size of the largest adenoma. In 75 cases adenomas were tubular only, while 72 had at least one villous or tubulovillous adenoma.

**Table II** Comparison of socio-demographic characteristics between (a) subjects with adenomas and (b) their FOB negative controls and (c) subjects who were FOB positive in whom no adenomas or carcinomas were found

| *Socio-demographic characteristics* | *(a) Cases with adenomas n (%)* | *(b) FOB negative controls for (a) n (%)* | *(c) FOB positive subjects, no adenoma or carcinoma n (%)* |
|---|---|---|---|
| *Total* | 147 | 153 | 176 |
| *Sex:* Male | 91 (62) | 94 (61) | 86 (49) |
| Female | 56 (38) | 59 (39) | 90 (51) |
| $\chi^2$, 3 d.f. | | (a)*vs*(b) 0.01, $P = 0.93$ | (a)*vs*(c) 5.50, $P = 0.02$ |
| *Age at interview* (years) | | | |
| 50–54 | 13 (9) | 14 (9) | 23 (13) |
| 55–59 | 26 (18) | 24 (16) | 37 (21) |
| 60–64 | 22 (15) | 26 (17) | 41 (23) |
| 65–69 | 37 (25) | 35 (23) | 35 (20) |
| 70–74 | 35 (24) | 40 (26) | 31 (18) |
| 75+ | 14 (10) | 14 (9) | 9 (5) |
| $\chi^2$, 5 d.f. | | (a)*vs*(b) 0.72, $P = 0.98$ | (a)*vs*(c) 9.29, $P = 0.10$ |
| Mean (±s.d.) | | | |
| Both sexes | 66 (7) | 66 (7) | 63 (7) |
| Male | 65 (7) | 65 (7) | 63 (7) |
| Female | 67 (8) | 68 (7) | 64 (8) |
| *Social class of longest occupation* (other than housewife) | | | |
| I | 11 (8) | 6 (4) | 8 (5) |
| II | 16 (11) | 29 (19) | 24 (14) |
| IIIN | 30 (20) | 41 (27) | 38 (22) |
| IIIM | 54 (37) | 55 (36) | 65 (37) |
| IV | 25 (17) | 13 (9) | 30 (17) |
| V | 3 (2) | 3 (2) | 4 (2) |
| Other (armed services, unemployed, retired, not in occupation due to war) | 8 (5) | 6 (4) | 7 (4) |
| $\chi^2$, 6 d.f. | | (a)*vs*(b) 10.90, $P = 0.09$ | (a)*vs*(c) 2.11, $P = 0.91$ |

### *Diet*

The estimated crude daily intakes of energy, fat, protein and fibre and subtypes of the latter, are presented in Table III. The median proportion of energy intake from fat ranged from 32.2% in the first quintile to 47.5% in the fifth quintile for cases and FOB negative controls combined; for cases and FOB positive controls, medians were 30.3% and 47.5% respectively. For total protein, the medians were 10.4% and 15.7% for cases and FOB negative controls combined, 10.6% and 16.8% for cases and FOB positive controls. For cases and FOB-negative controls combined, the median intake of energy from carbohydrate was 38% in the first quintile and 54% in the highest quintile; similar values were found for cases and FOB-positive controls combined. The distributions of retinol and vitamin C were non-normal; in subsequent analysis, a logarithmic transformation was applied.

### *Fat*

As shown in Table IV, no association was found with reported intake of total fat, saturated fat or monounsaturated fat, after adjustment for age, sex, social class and total energy intake. In the comparison with FOB-negative controls, an unexpected significant positive association with polyunsaturated fat was found. This positive association was also evident in a matched analysis and when separate analyses were carried out for men and woman. By contrast, a reduced risk of adenomas was associated with the upper four quintiles of intake in the comparison with the other control group; there was no significant trend. This positive association with polyunsaturated fat was found for all subgroups of cases except those with small adenomas only.

### *Protein*

No association with total protein intake, protein of vegetable or animal origin or specifically from meat, was found in the comparison with FOB-negative controls. A statistically significant inverse association with total protein intake was found in the comparison with the other control group (Table IV). This inverse association was also apparent when separate analyses for each sex were carried out, and remained statistically significant for men. The inverse association with total protein found in the comparison with FOB-positive subjects was apparent for all of the subgroups of cases.

The inverse association apparent in the comparisons with FOB-positive subjects was also found for protein from animal sources (chi-square for trend = 5.07, $P = 0.024$). No significant association with protein from vegetable sources was found, although the relative risk for the highest quintile of intake was 0.5. No association with protein from meat was found.

As FOB-positive controls with symptomatic diverticular disease (12 subjects), inflammatory bowel disease (12 subjects) or coeliac disease (one subject) might have altered their diet, we repeated the analysis with these subjects excluded but this had little effect.

### *Total fibre*

In the comparison with FOB-negative controls the crude RR for the highest versus the lowest quintile was 0.6 but the relationship was non-linear. No association with intake or total fibre was apparent after adjustment for age, sex, social class and energy intake (Table V) or in a matched analysis.

In the comparison with FOB-positive controls, an inverse association was also found, but this was not statistically significant either in the crude analysis or after adjustment for age, sex and social class. The relative risk estimates did not change markedly when FOB-positive controls with symptomatic diverticular disease, inflammatory bowel disease or coeliac disease were excluded.

### *Cereal fibre*

No clear association with cereal fibre was found in the comparison with FOB-negative controls, although the adjusted RR for the highest quintile of intake was 0.6 (Table V). However, in the comparison with FOB-positive subjects, a strong inverse association was found. Adjustment for energy intake had little effect on the RRs, and increased the value of the trend statistic. The inverse association was ap-

**Table III** Distribution of estimated daily intake of specific nutrients by status and sex

| | Median values | | | | | | | |
|---|---|---|---|---|---|---|---|---|
| | Men | | | Women | | | Cases and FOB-negative controls combined[a] | |
| Nutrient | Cases | FOB negative controls | FOB positive subjects | Cases | FOB negative controls | FOB positive subjects | 1st quintile | 5th quintile |
| Energy (kcal) | 2293 | 2356 | 2240 | 1849 | 1914 | 1869 | 1573 | 2941 |
| Total fat (g)[b] | 95 | 102 | 90 | 89 | 86 | 79 | 62 | 136 |
| Saturated fat (g) | 33 | 34 | 31 | 30 | 33 | 27 | 20 | 55 |
| Monounsaturated fat (g) | 28 | 29 | 27 | 26 | 24 | 23 | 17 | 44 |
| Polyunsaturated fat (g) | 13 | 14 | 14 | 12 | 10 | 11 | 7 | 22 |
| Total protein (g)[b] | 72 | 74 | 73 | 60 | 61 | 63 | 49 | 93 |
| from animal sources (g) | 32 | 33 | 33 | 27 | 29 | 29 | 19 | 47 |
| from meat (g) | 10 | 10 | 9 | 9 | 8 | 9 | 4[c] | 17 |
| from vegetable sources (g) | 29 | 30 | 30 | 26 | 24 | 26 | 19 | 38 |
| Total fibre (g) | 24 | 26 | 27 | 27 | 24 | 27 | 16 | 38 |
| Cereal fibre (g) | 6 | 8 | 9 | 7 | 8 | 8 | 4 | 15 |
| Cholesterol (mg) | 331 | 342 | 328 | 307 | 320 | 298 | 182 | 505 |
| Cruciferous vegetables (g) | 40 | 43 | 42 | 49 | 43 | 42 | 17[d] | 80 |
| Calcium (mg) | 763 | 781 | 776 | 669 | 683 | 666 | 484 | 1073 |
| Phosphorous (mg) | 1197 | 1310 | 1300 | 1098 | 1081 | 1135 | 851 | 1614 |
| Vitamin D (μg) | 3 | 3 | 4 | 3 | 3 | 3 | 1 | 7 |
| Carbohydrate (g) | 257 | 283 | 273 | 223 | 216 | 227 | 175 | 349 |
| Retinol (μg) | 567 | 722 | 609 | 540 | 623 | 574 | 290 | 2656 |
| Carotene (μg) | 3471 | 3678 | 3789 | 3899 | 3266 | 3597 | 1806 | 8014 |
| Vitamin C (mg) | 75 | 82 | 93 | 109 | 101 | 102 | 44 | 154 |
| Vitamin E (mg) | 37 | 38 | 38 | 40 | 35 | 35 | 24 | 54 |
| Iron (mg) | 13 | 13 | 14 | 12 | 11 | 12 | 9 | 17 |

[a]The medians of the first and fifth quintiles of cases and FOB positive subjects are not presented as they are similar to those for cases and FOB negative controls combined. [b]The distribution of fat and protein, and subtypes of these, have not been adjusted for energy in this tabulation. [c]One case, one FOB negative control and three FOB positive subjects reported that they did not eat meat. These subjects were allocated to a separate category; the quintiles relate to consumers. [d]One FOB negative control and two FOB positive subjects reported that they did not eat cruciferous vegetables. These subjects were allocated to a separate category; the quintiles relate to consumers.

parent for men and women when separate analyses for each sex were carried out, and remained statistically significant for men. The RRs were changed little by exclusion of subjects with symptomatic diverticular disease, inflammatory bowel disease or coeliac disease, and the trend remained significant ($\chi^2 = 5.24$, $P = 0.022$). The inverse association was apparent for all subgroups except for cases with large adenomas and was statistically significant for medium adenomas, small adenomas, and tubular adenomas.

In the comparison with FOB positive subjects, the inverse association with total protein was diminished and no longer statistically significant, and that with protein from vegetable sources was no longer apparent, when cereal fibre was included in the model. The inverse associations with animal protein and with cereal fibre remained statistically significant when both were included in the model; no interaction was found.

### *Calcium and related nutrients*

No association was found with reported intake of calcium or phosphorous. In the comparison with FOB negative controls only, a positive association with vitamin D was found ($\chi^2$ for trend = 4.38, $P = 0.04$, after adjustment for age, sex, social class, total energy intake and year of notification); the association was no longer apparent when polyunsaturated fat was included in the model.

### *Other nutrients*

No association was found with reported intake of cholesterol, retinol, carotene, vitamin E, carbohydrate, cruciferous vegetables or iron. In the comparison with FOB positive subjects only, an inverse association was found with vitamin C ($\chi^2$ for trend = 5.77, $P = 0.02$, after adjustment for age, sex, social class, year of notification and year of interview). This association did not persist after adjusting for intake of cereal fibre.

### *Frequency of consumption of the 'marker' foods*

There was no association between adenomas and frequency of eating beef, chicken, fish, biscuits, yoghurt or fruit. This was found also for cheese, except that subjects who ate these less than once a month were at an increased risk compared with more frequent consumers. Compared with subjects eating cheese more often than once a month the RR was 2.8 (95% confidence interval 1.1–7.1) in the comparison with FOB negative controls and 2.3 (95% confidence interval 1.0–5.1) in the comparison with the other group.

### *Markers of fat intake*

In the comparison with FOB negative controls, there was no difference in risk between those who preferred mainly grilled foods, mainly fried foods, or those who indicated no particular preference. However, in the comparisons with FOB positive subjects, the RR was 2.1 (95% CI 1.1–4.4) for subjects whose preference was mainly for fried food, and 1.7 (95% CI 0.95–3.2) for those with no particular preference. Amongst married subjects, there was no increase in risk in subjects who reported that they ate more fat than their spouse. In the questions about how much fat subjects ate on different meats, the risk of adenomas in subjects who reported that they ate no fat were similar to those of subjects who reported that they ate most or all the fat on each of the meats considered.

**Table IV** Associations between adenomas and reported intake of total fat, subtypes of fat and total protein

| | Comparison with | | | | | |
|---|---|---|---|---|---|---|
| | *FOB-negative controls* | | | *FOB-positive subjects* | | |
| | *Number of:* | | | *Number of:* | | |
| *Quintile of intake* | *Cases* | *Controls* | *RR (95% CI)* | *Cases* | *Controls* | *RR (95% CI)* |
| *Total fat* | | | | | | |
| 1st | 29 | 31 | 1.0 | 26 | 39 | 1.0 |
| 2nd | 28 | 32 | 0.86 (0.41–1.83) | 30 | 34 | 1.48 (0.70–3.12) |
| 3rd | 31 | 29 | 1.11 (0.53–2.35) | 31 | 34 | 1.61 (0.77–3.39) |
| 4th | 29 | 31 | 1.01 (0.47–2.14) | 27 | 37 | 1.06 (0.50–2.25) |
| 5th | 30 | 30 | 1.06 (0.50–2.24) | 33 | 32 | 1.68 (0.80–3.55) |
| Chi-square for trend | | | 0.10, $P = 0.752$ | | | 0.68, $P = 0.411$ |
| *Saturated fat* | | | | | | |
| 1st | 33 | 27 | 1.0 | 31 | 34 | 1.0 |
| 2nd | 30 | 30 | 0.86 (0.41–1.80) | 24 | 40 | 0.69 (0.33–1.43) |
| 3rd | 21 | 39 | 0.46 (0.21–0.97) | 26 | 39 | 0.83 (0.40–1.74) |
| 4th | 33 | 27 | 0.98 (0.46–2.08) | 30 | 34 | 0.99 (0.47–2.05) |
| 5th | 30 | 30 | 0.87 (0.41–1.84) | 36 | 29 | 1.40 (0.68–2.92) |
| Chi-square for trend | | | 0.04, $P = 0.843$ | | | 1.53, $P = 0.215$ |
| *Monounsaturated fat* | | | | | | |
| 1st | 28 | 32 | 1.0 | 32 | 33 | 1.0 |
| 2nd | 32 | 28 | 1.33 (0.63–2.79) | 28 | 36 | 0.83 (0.40–1.71) |
| 3rd | 25 | 35 | 0.83 (0.39–1.76) | 25 | 40 | 0.66 (0.32–1.38) |
| 4th | 30 | 30 | 1.17 (0.55–2.49) | 29 | 35 | 0.92 (0.44–1.92) |
| 5th | 32 | 28 | 1.39 (0.66–2.91) | 33 | 32 | 0.96 (0.47–1.97) |
| Chi-square for trend | | | 0.41, $P = 0.524$ | | | 0.00, $P = 0.990$ |
| *Polyunsaturated fat* | | | | | | |
| 1st | 21 | 39 | 1.0 | 40 | 25 | 1.0 |
| 2nd | 34 | 26 | 2.81 (1.31–6.06) | 24 | 40 | 0.38 (0.18–0.81) |
| 3rd | 25 | 35 | 1.58 (0.74–3.42) | 20 | 45 | 0.26 (0.12–0.56) |
| 4th | 36 | 24 | 3.47 (1.60–7.54) | 33 | 31 | 0.65 (0.31–1.38) |
| 5th | 31 | 29 | 2.34 (1.09–5.03) | 30 | 35 | 0.48 (0.23–1.00) |
| Chi-square for trend | | | 4.78, $P = 0.029$ | | | 1.09[a], n.s. |
| *Total protein* | | | | | | |
| 1st | 27 | 33 | 1.0 | 38 | 27 | 1.0 |
| 2nd | 28 | 32 | 1.14 (0.54–2.42) | 28 | 36 | 0.47 (0.22–0.98) |
| 3rd | 35 | 25 | 1.76 (0.84–3.71) | 36 | 29 | 0.88 (0.42–1.83) |
| 4th | 30 | 30 | 1.22 (0.57–2.59) | 19 | 45 | 0.25 (0.11–0.53) |
| 5th | 27 | 33 | 1.08 (0.50–2.29) | 26 | 39 | 0.46 (0.22–0.97) |
| Chi-square for trend | | | 0.05, $P = 0.819$ | | | 6.11, $P = 0.013$ |

[a]The model in which the five quintiles of intake of polyunsaturated fat were treated as levels of an ordinal variable provided a poor fit to the data. Exclusion of one subject resulted in an adequate fit but had little effect on the value of the chi-square for trend.

**Table V** Associations between adenomas and reported intake of total fibre and cereal fibre

| | Comparison with | | | | | |
|---|---|---|---|---|---|---|
| | *FOB-negative controls* | | | *FOB-positive subjects* | | |
| | *Number of:* | | *RR adjusted*[b] | *Number of:* | | *RR adjusted*[c] |
| *Quintile of intake* | *Cases* | *Controls* | *(95% CI)* | *Cases* | *Controls* | *(95% CI)* |
| *Total fibre*[a] | | | | | | |
| 1st | 33 | 27 | 1.0 | 34 | 31 | 1.0 |
| 2nd | 25 | 35 | 0.69 (0.32–1.45) | 34 | 30 | 1.08 (0.53–2.23) |
| 3rd | 34 | 26 | 1.16 (0.54–2.49) | 25 | 40 | 0.58 (0.28–1.21) |
| 4th | 29 | 31 | 1.01 (0.47–2.17) | 28 | 36 | 0.88 (0.42–1.82) |
| 5th | 26 | 34 | 0.81 (0.37–1.78) | 26 | 39 | 0.63 (0.30–1.31) |
| Chi-square for trend | | | 0.0, n.s. | | | 1.86, n.s. |
| *Cereal fibre*[d,e] | | | | | | |
| 1st | 30 | 30 | 1.0 | 49 | 38 | 1.0 |
| 2nd | 29 | 31 | 0.99 (0.46–2.12) | 20 | 21 | 0.74 (0.34–1.60) |
| 3rd | 28 | 26 | 1.71 (0.80–3.67) | 28 | 38 | 0.54 (0.27–1.09) |
| 4th | 33 | 30 | 1.23 (0.58–2.63) | 33 | 34 | 0.73 (0.37–1.42) |
| 5th | 17 | 36 | 0.57 (0.25–1.29) | 17 | 43 | 0.29 (0.14–0.59) |
| Chi-square for trend | | | 0.55, n.s. | | | 8.80, $P = 0.003$[c] |

[a]The cut points between quintiles in the comparison with FOB negative controls were 18.4, 23.4, 28.0 and 33.9 g day, and in the comparison with FOB positive subjects they were 19.2, 24.5, 28.3 and 33.9 g day. [b]Age, sex, social class and total energy intake. [c]Adjusted for age, sex, social class and interactions between age and sex, age and social class. [d]The cut points between quintiles in the comparison with FOB negative controls were 4.2, 5.7, 9.2 and 11.8 g day, and in the comparison with FOB positive subjects, they were 4.7, 6.6, 9.2 and 11.8 g day. [e]Two FOB positive subjects reported that they did not consume any cereal fibre; they have been excluded from the analysis.

### Discussion

The results of the present study do not support the hypothesis that the risk of developing colorectal adenomas increases with increasing intake of animal fat or protein. No association with total fat, saturated fat or monounsaturated fat was found in comparison with either control group. There was no evidence of a positive association with total protein or specific sources of protein; indeed, a significant *inverse* association with both total protein and protein from animal sources was found in the comparison with FOB-positive subjects. A significant inverse association with cereal fibre was apparent in the comparison with FOB-positive subjects. There was a significant positive association with polyunsaturated fat in the comparison with FOB-negative controls.

Before comparing these findings with those of previous studies, we first consider certain aspects of the design and methods of the present study. One of the strengths of the present study is that it relates to subjects with asymptomatic adenomatous polyps. In many previous studies, subjects with colorectal adenoma have been identified as a result of gastrointestinal symptoms which in the majority are unrelated to the presence of the adenomas and are probably functional. The proportion of cases with small adenomas is likely to have been substantially lower in the present study that in other studies. For example, the proportion of cases whose largest adenoma was less than 1 cm in maximum diameter was 30% in the present study, in contrast to 66% in the study of Macquart-Moulin *et al.* (1987). Therefore, our study should have had greater power to detect associations with large adenomas, which are the most likely to be associated with malignant changes (O'Brien *et al.*, 1990; Gatteschi *et al.*, 1991; Chantereau *et al.*, 1992).

Control subjects were recruited among participants in a trial of FOB screening. Thus, the study was free of the selection bias arising with use of hospital controls. Selection bias associated with compliance with screening should have affected case and control groups equally. It is likely that a proportion of the FOB negative control subjects had adenomas. Inclusion of the second control group, comprising FOB positive subjects in whom no adenoma or carcinoma was found was specifically for the purpose of assessing the consistency of association in comparison with groups in whom polyps could be excluded with a high degree of certainty and one in which the presence of polyps could not be excluded. One possible explanation for the inconsistency of some findings in comparison with the two sets of controls is that FOB positive subjects in whom no adenoma or carcinoma was found were atypical of the general population. For most of these subjects, the reason for the positive test result was not apparent. In an earlier study, the proportion of these subjects who had upper gastrointestinal symptoms at the time they completed the FOB test was low, and in follow-up of 269 subjects free of these symptoms for a median period of 5 years, only five were referred for investigation of symptoms which had developed since the patients were screened, all of whom had benign upper gastrointestinal conditions (Thomas & Hardcastle, 1990). In the present study, 12 (7%) of these subjects had inflammatory bowel disease and 12 had diverticular disease.

The difficulties of assessing past diet are well known. Nevertheless, the correlation coefficients for intakes of specific nutrients reported at the original and repeat interviews (Table I) were similar to those reported in other studies (Willett,, 1989*b*, p96). Elsewhere, we have shown that the agreement between the diet history interview used in the present study and a validated self-completed questionnaire for intakes of total fat, fibre and calcium was similar to that in previous studies (Jackson *et al.*, 1990). In addition, only three subjects volunteered that they had changed their diet since notification of the test result, one FOB negative control and two FOB positive subjects.

The sole positive association found was for polyunsaturated fat in the comparison with FOB negative controls. No such association has been found in the other studies in which

**Table VI** Summary of results of studies of associations between colorectal polyps and nutrient intakes

| | Present study[a] | | Esser *et al.*, 1980 | Hoff *et al.*, 1986[b] | | Macquart-Moulin *et al.*, 1987 | Stemmermann *et al.*, 1988 | Neugut *et al.*, 1990[c] | | Kune *et al.*, 1991 | Giovannucci *et al.*, 1992 |
|---|---|---|---|---|---|---|---|---|---|---|---|
| *Nutrient* | *I* | *II* | | *I* | *II* | | | *I* | *II* | | |
| Total fat | 0 | 0 | 0 | 0 | + | 0 | 0 | 0 | 0 | 0 | + |
| Saturated | 0 | 0 | 0 | 0 | 0 | + | 0 | 0 | + | | + |
| Monounsaturated | 0 | 0 | | | | 0 | 0 | | | | + |
| Polyunsaturated | + | 0 | | | | 0 | 0 | | | | 0 |
| Total protein | 0 | – | 0 | | | + | 0 | 0 | 0 | | 0 |
| Vegetable | 0 | – | | | | – | | | | | |
| Animal | 0 | – | | 0 | 0 | 0 | 0 | | | | |
| Meat | 0 | 0 | | | | 0 | | | | | 0 |
| Total fibre | 0 | 0 | 0 | 0 | – | – | | 0 | – | – | – |
| Cereal fibre | 0 | – | 0 | | | 0 | | | | | |

Note: 0, no association; +, positive association ; –, inverse association. [a]I, comparison with FOB-negative controls; II, comparison with FOB-positive subjects. [b]I, cases with polyps less than 5 mm; II, cases with adenomas 5 mm or larger. [c]I, men; II, women.

it has been considered (Macquart-Moulin *et al.*, 1987; Giovannucci *et al.*, 1992) and it is quite possible that this is a chance finding.

Inverse associations were found with total protein, both animal and vegetable protein sources, and with cereal fibre, but only in comparison with the FOB positive controls. The association with protein from vegetable sources was no longer apparent when cereal fibre was included in the model, reflecting the high correlation ($r = 0.61$) between intakes of the two nutrients. The inverse associations with animal protein and cereal fibre remained significant when both were included in the model. The association with protein is difficult to interpret as there is no evidence of any relation in comparison with the FOB negative controls, whereas the lower relative risk associated with the highest quintile of cereal fibre intake is apparent in the comparison with both control groups. The inverse associations apparent in the comparison with FOB positive controls remained when 25 subjects with symptomatic inflammatory bowel disease, diverticular disease or coeliac disease at the time of screening were excluded.

Our results are compared with those of previous studies relating to nutrient intakes in Table VI. No consistent pattern has been found and the comparison is complicated by substantial differences in the definition and methods of ascertainment of cases, the nature of the control group considered, the nutrients considered and the methods of assessing dietary intake and the methods of statistical analysis.

Only in two studies has a significant association with total fat been found (Hoff *et al.*, 1986; Giovanucci *et al.*, 1992). However, in the analysis of Hoff *et al.*, fat intake was assessed as a percentage of total energy intake (the nutrient density technique). The energy intake of cases with adenomas 5 mm or larger was substantially lower than that of controls. Hence, the positive association with total fat found in their study may be an artifact, as nutrient densities tend to be associated with disease in the direction opposite to that of total energy intake (Willett, 1989*b* pp. 258–261). The association with total fat found in the study of Giovannucci *et al.* is largely accounted for by saturated fat. Positive associations with this nutrient have also been found in two other studies (Macquart-Moulin *et al.*, 1987; Neugut *et al.*, 1990) although in both studies some or all cases were symptomatic.

With regard to protein intake our finding of no association is consistent with all but one (Macquart-Moulin *et al.*, 1987) of the previous studies. Lack of, or inverse association with fat and protein is consistent with a large Japanese study in which frequency of consumption of certain food groups were compared between cases with symptomatic adenomas and population-based controls (Kato *et al.*, 1990).

The associations with total fibre intake have been inconsistent. The median intake of fibre was somewhat higher than that reported in the studies of Macquart-Moulin *et al.* (1987) and Giovannucci *et al.* (1992), in both of which an inverse association with high fibre intake was found, but the variability of intake was similar between the studies. Willett (1989*a*) observed that in all eight case-control studies of colorectal cancer in which the source of fibre were examined separately, grain fibre or cereal intake was either unrelated or positively associated with the disease, whilst intake of fibre from fruits and vegetables was protective, an effect also observed in two additional case-control studies. He suggested that agents other than specific fractions of fibre might account for this protective effect. In our study, the association with protein from vegetable sources and vitamin C apparent in the comparison with FOB-positive controls only was no longer apparent when cereal fibre was included in the model. No association with retinol, carotene, vitamin E or cruciferous vegetables was found, in general agreement with previous studies (Hoff *et al.*, 1986; Macquart-Moulin *et al.*, 1987; Neugut *et al.*, 1988, 1990). No association with frequency of consumption of fresh fruit was found. Thus, our finding of an inverse association with cereal fibre in the comparison with FOB-positive subjects cannot be attributed to fruits and vegetables.

### Conclusion

So far, studies of diet and colorectal adenomas have not provided consistent evidence of increasing risk with increasing intake of animal fat or protein, or of protective effects of dietary fibre or calcium. It is not clear how far this reflects differences in study methods and in particular the difficulty of assessing diet, or how far this reflects the low malignant potential of colorectal adenomas as ascertained in these studies.

We gratefully acknowledge financial support from the Cancer Research Campaign. We thank the subjects who participated in the study and their general practitioners for their co-operation. We are most grateful for Helen Crawley's contribution in reviewing the portion size estimates. In Nottingham, we thank Gwyn Campion and Jenny Sterland for a major contribution to the interviewing, Mary Stevenson for her help with data processing, Chris Mangham and Jane Jackson for administrative assistance, and Janice Gillard for secretarial help. In Lyon, we thank Patrick Maisonneuve for advice on the analysis, Dr Elio Riboli for helpful comments, and Sheila Stallard and Jill Rawling for their ever-patient secretarial assistance.

### References

ARMITAGE, N., HARDCASTLE, J.D., AMAR, S.S., BALFOUR, T.W., HAYNES, J. & JAMES, P.D. (1985). A comparison of an immunological faecal occult blood test Fecatwin sensitive/FECA EIA with Haemoccult in population screening for colorectal cancer. *Br. J. Cancer*, **51**, 799–804.

BAKER, R.J., CLARKE, M.R.B. & NELDER, J.A. (1985). *GLIM: The Generalised Linear Interactive Modelling System*. GLIM 3.77 Manual and Macro Library Release 1.1 Oxford: Numerical Algorithms Group.

BURKITT, D.P. (1971). Epidemiology of cancer of the colon and rectum. *Cancer*, **28**, 3–13.

CHANTEREAU, M.J., FAIVRE, J., BOUTRON, M.C., PIARD, F., ARVEUX, P., BEDENNE, L. & HILLON, P. (1992). Epidemiology, management, and prognosis of malignant large bowel polyps within a defined population. *Gut*, **33**, 259–263.

DRASAR, B.S. & IRVING, D. (1973). Environmental factors and cancer of the colon and breast. *Br. J. Cancer*, **27**, 167–172.

ESSER, W., WEITHOFER, G. & BLOCH, R. (1980). The significance of dietary fat and fiber for the aetiology of colon cancer. *Z. Gastroenterologie*, **18**, 30–37.

GATTESCHI, B., COSTANTINI, M., BRUZZI, P., MERLO, F., TORCOLI, R. & NICOLO, G. (1991). Univariate and multivariate analyses of the relationship between adenocarcinoma and solitary and multiple adenomas in colorectal adenoma patients. *Int. J. Cancer*, **49**, 509–512.

GIOVANNUCCI, E., STAMPFER, M.J., COLDITZ, G., RIMM, E.B. & WILLETT, W.C. (1992). Relation of diet to risk of colorectal adenoma in men. *JNCI*, **84**, 91–98.

HARDCASTLE, J.D., THOMAS, W.M., CHAMBERLAIN, J., PYE, G., SHEFFIELD, J., JAMES, P.D., BALFOUR, T.W., AMAR, S.S., ARMITAGE, N.C. & MOSS, S.M. (1989). Randomised, controlled trial of faecal occult blood screening for colorectal cancer. Results for first 107,349 subjects. *Lancet*, **i**, 1160–1164.

HOFF, G., MOEN, I.E., TRYGG, K., FRØLICH, W., SAUAR, J., VATN, M., GJONE, E. & LARSEN, S. (1986). Epidemiology of polyps in the rectum and sigmoid colon. Evaluation of nutritional factors. *Scand. J. Gastroenterol.*, **21**, 199–204.

HOSMER, D.W. & LEMESHOW, S. (1989). *Applied Logistic Regression*. New York: Wiley.

HSIEH, C.-C., MAISONNEUVE, P., BOYLE, P., MACFARLANE, G.J. & ROBERTSON, C. (1991). Analysis of quantitative data by quantiles in epidemiologic studies: classification according to cases, noncases, or all subjects? *Epidemiology*, **2**, 137–140.

JACKSON, N., LITTLE, J. & WILSON, A.D. (1990). Comparison of a diet history interview and a self completed questionnaire in assessment of diet in an elderly population. *J. Epidemiol. Community Health*, **44**, 162–169.

KATO, I., TOMINAGA, S., MATSUURA, A., YOSHII, Y., SHIRAI, M. & KOBAYASHI, S. (1990). A comparative case-control study of colorectal cancer and adenoma. *Jpn. J. Cancer Res.* **81**, 1101–1108.

KUNE, A., KUNE, S., READ, A., MACGOWAN, K., PENFOLD, C. & WATSON, L.F. (1991). Colorectal polyps, diet, alcohol, and family history of colorectal cancer: a case-control study. *Nutr. Cancer*, **16**, 25–30.

MACFARLANE, G.J., BOYLE, P. & MAISONNEUVE, P. (1991). *SEARCH: A computer package to assist the statistical analysis of case-control studies*. International Agency for Research on Cancer: Lyons.

MACQUART-MOULIN, G., RIBOLI, E., CORNEE, J., KAAKS, R. & BERTHEZENE, P. (1987). Colorectal polyps and diet: A case-control study in Marseilles. *Int. J. Cancer*, **40**, 179–188.

NATIONAL ADVISORY COMMITTEE ON NUTRITIONAL EDUCATION (1983). *A Discussion Paper on Proposals for Nutritional Guidelines for Health Education in Britain*. Health Education Council: London.

NEUGUT, A.I., JOHNSEN, C.M., FORDE, K.A., TREAT, M.R. & NIMS, C. (1988). Vitamin supplements among women with adenomatous polyps and cancer of the colon. *Dis. Colon Rectum*, **31**, 430–432.

NEUGUT, A.I., GARBOWSKI, G., NIEVES, J., MURRAY, T., FORDE, K.A., WAYE, J., TREAT, M.R. & FENOGLIO-PREISER, C. (1990). Diet and colorectal adenomatous polyps: a case-control study. *Am. J. Epidemiol.*, **132**, 783–784.

NEWMARK, H.L., WARGOVICH, M.J. & BRUCE, W.R. (1984). Colon cancer and dietary fat, phosphate, and calcium: a hypothesis. *JNCI*, **72**, 1323–1325.

O'BRIEN, M.J., WINAWER, S.J., ZAUBER, A.J., GOTTLIEB, L.S., STERNBERG, S.S., DIAZ, B., DICKERSIN, G.R., EWING, S., GELLER, S., KASIMIAN, D., KOMOROWSKI, R., SZPORN, A. & THE NATIONAL POLYP STUDY WORKGROUP. (1990). The National Polyp Study. Patient and polyp characteristics associated with high-grade dysplasia in colorectal adenomas. *Gastroenterology*, **98**, 371–379.

PAUL, A.A. & SOUTHGATE, D.A.T. (1978). *McCance and Widdowson's "The Composition of Foods"*. 4th ed., HMSO: London.

ROTHMAN, K.J. (1986). *Modern Epidemiology*, pp 243–244. Little, Brown and Co.: Boston.

STEMMERMANN, G.N., HEILBRUN, L.K. & NOMURA, A.M.Y. (1988). Association of diet and other factors with adenomatous polyps of the large bowel: a prospective autopsy study. *Am. J. Clin. Nutr.*, **47**, 312–317.

THOMAS, W.M. & HARDCASTLE, J.D. (1990). Role of upper gastrointestinal investigations in a screening study for colorectal neoplasia. *Gut*, **31**, 1294–1297.

WILLETT, W. (1989*a*). The search for the causes of breast and colon cancer. *Nature*, **338**, 389–394.

WILLETT, W. (1989*b*). *Nutritional Epidemiology*. Oxford University Press: New York.

WILLETT, W. & STAMPFER, M.J. (1986). Total energy intake: implications for epidemiologic analyses. *Am. J. Epidemiol.*, **124**, 17–26.

WYNDER, E.L. & SHIGEMATSU, T. (1967). Environmental factors of cancer of the colon and rectum. *Cancer*, **20**, 1520–1561.

ZARIDZE, D.G. (1983). Environmental etiology of large-bowel cancer. *JNCI*, **70**, 389–400.


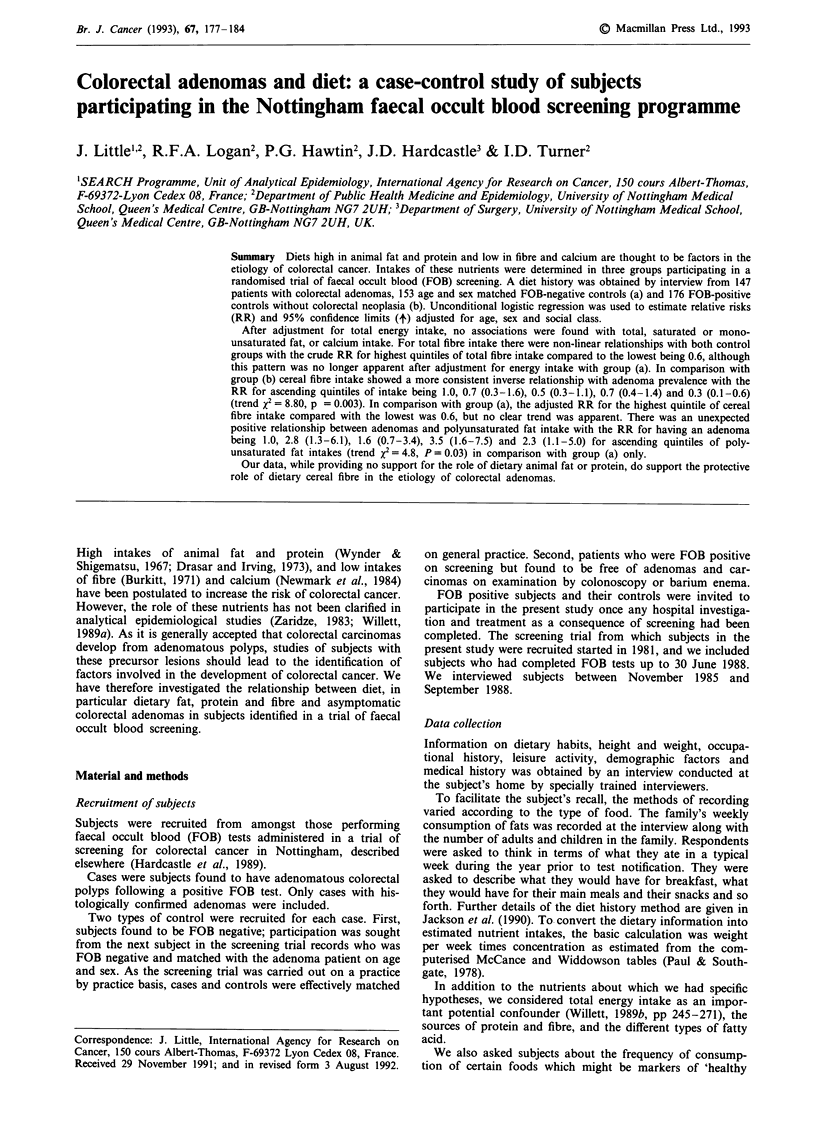

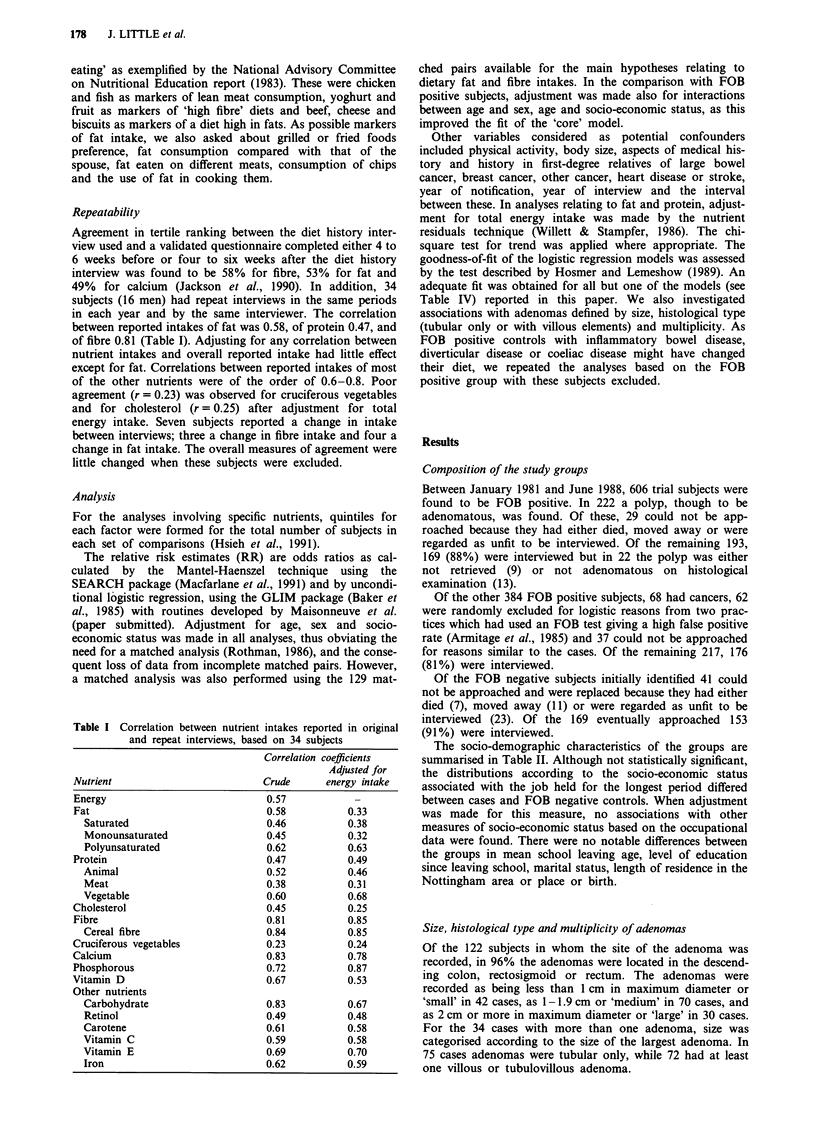

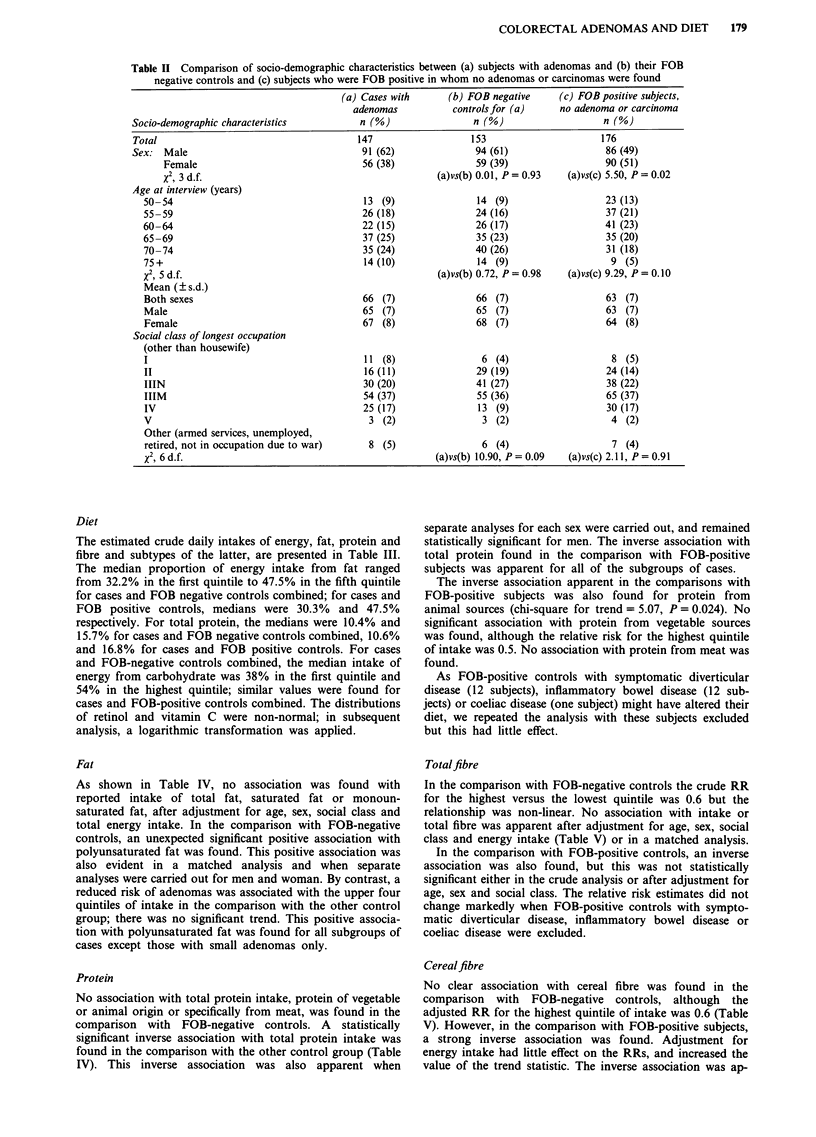

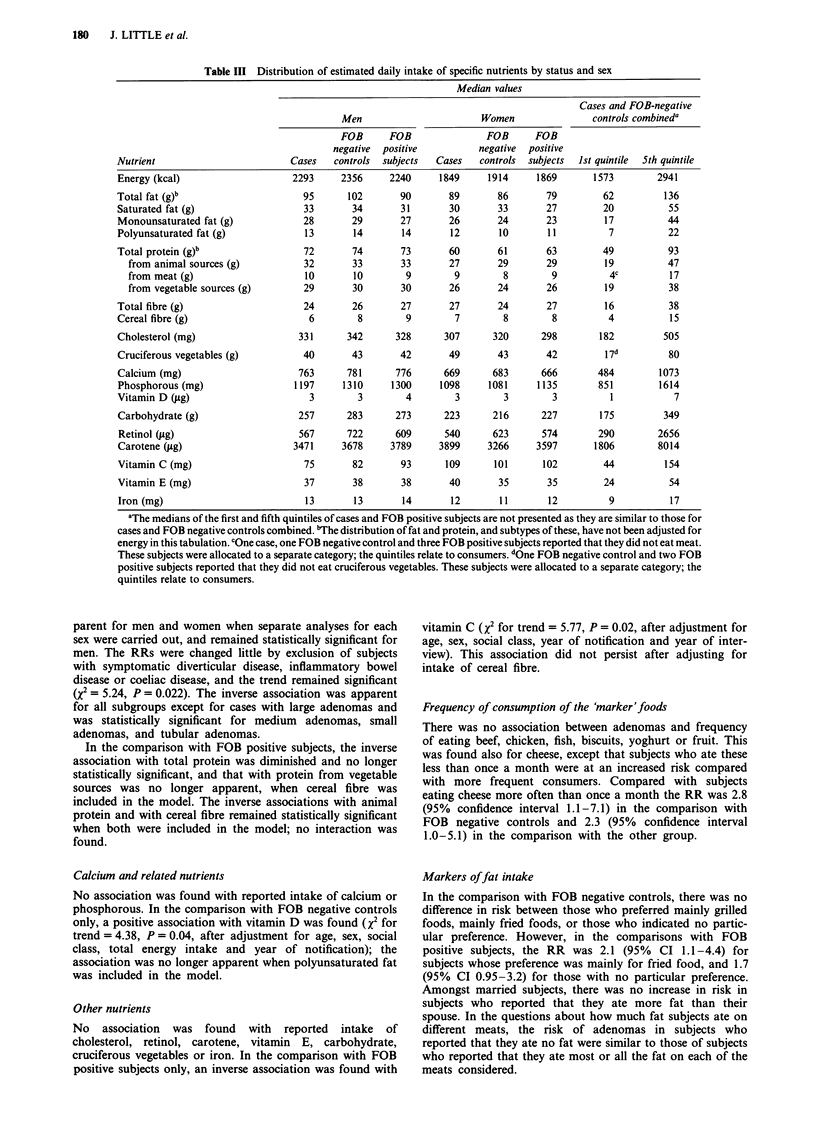

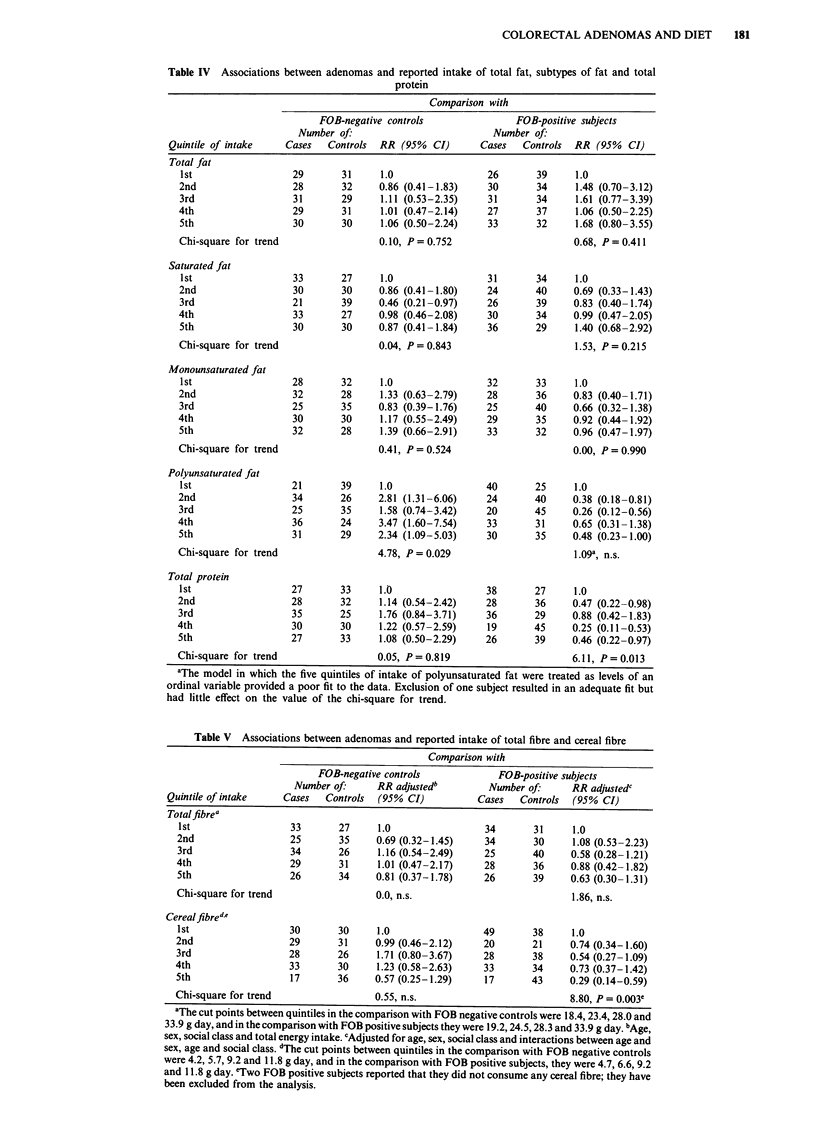

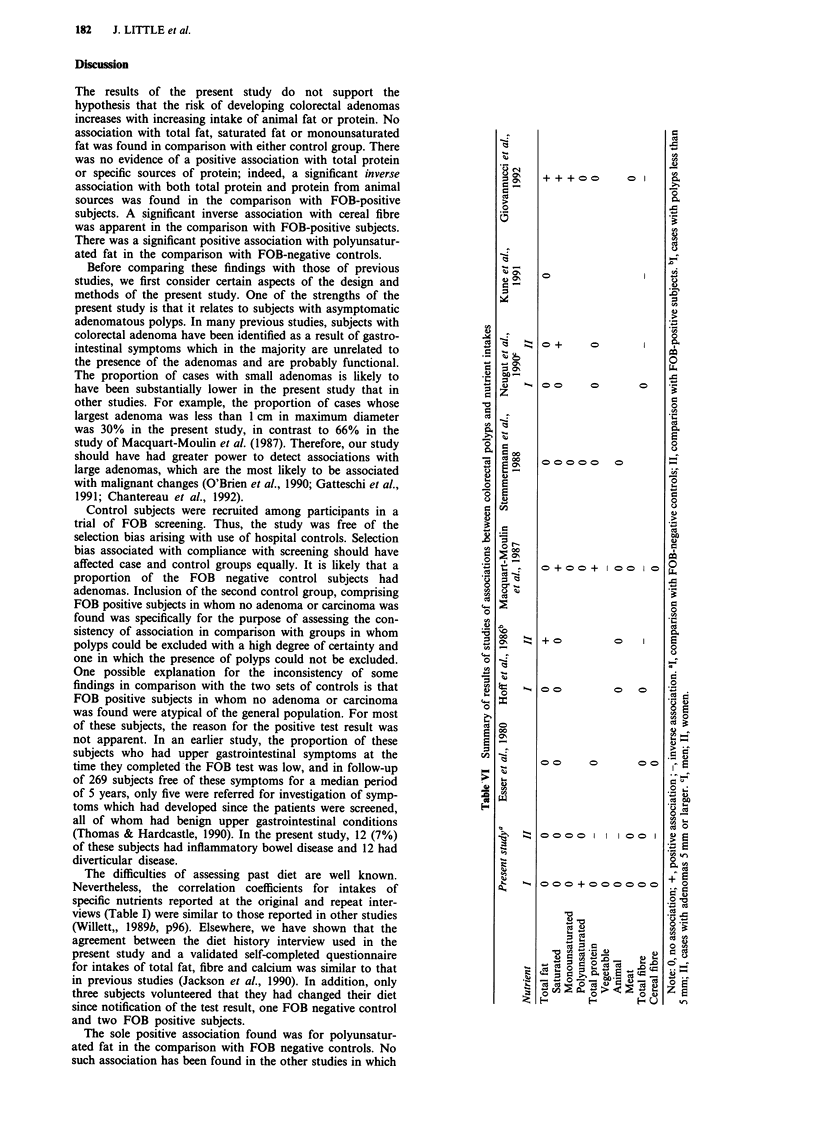

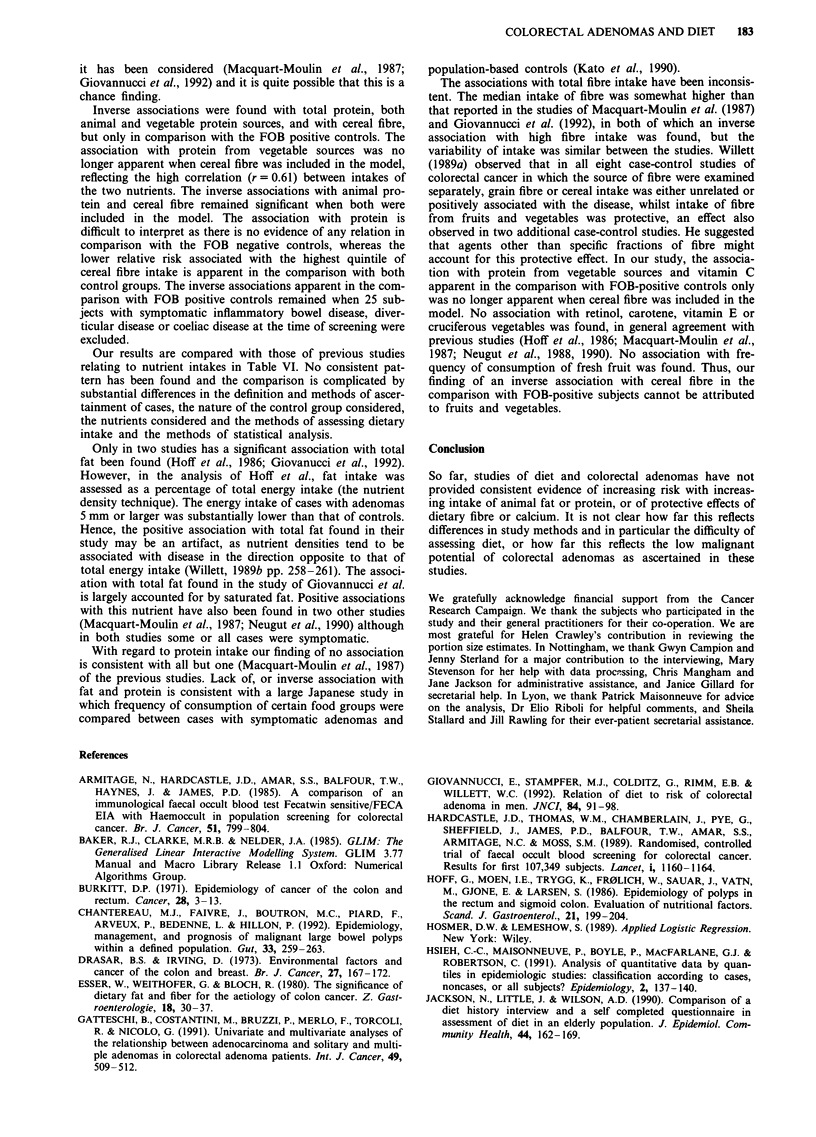

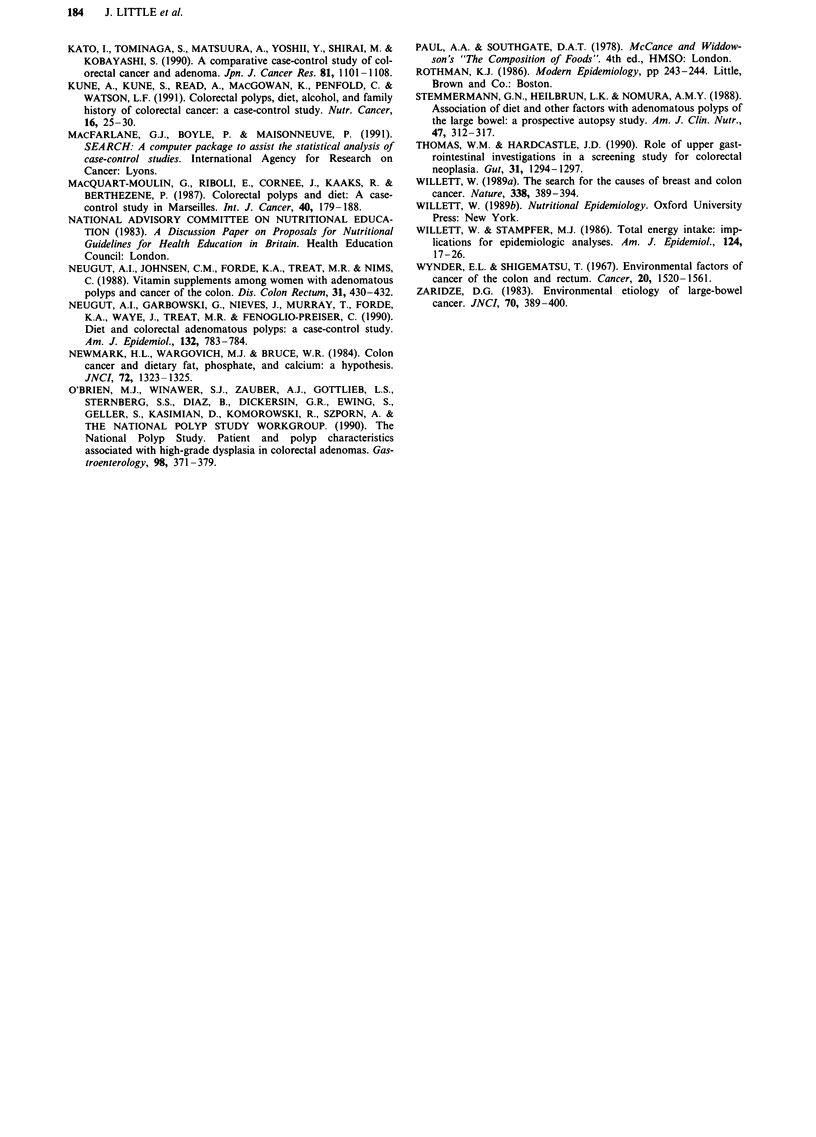


## References

[OCR_01373] Armitage N., Hardcastle J. D., Amar S. S., Balfour T. W., Haynes J., James P. D. (1985). A comparison of an immunological faecal occult blood test Fecatwin sensitive/FECA EIA with Haemoccult in population screening for colorectal cancer.. Br J Cancer.

[OCR_01386] Burkitt D. P. (1971). Epidemiology of cancer of the colon and rectum.. Cancer.

[OCR_01390] Chantereau M. J., Faivre J., Boutron M. C., Piard F., Arveux P., Bedenne L., Hillon P. (1992). Epidemiology, management, and prognosis of malignant large bowel polyps within a defined population.. Gut.

[OCR_01396] Drasar B. S., Irving D. (1973). Environmental factors and cancer of the colon and breast.. Br J Cancer.

[OCR_01400] Esser W., Weithofer G., Bloch R. (1980). Zur Bedeutung des Fett- und Rohfasergehalts der Nahrung für die Entstehung des Kolonkarzinoms.. Z Gastroenterol.

[OCR_01405] Gatteschi B., Costantini M., Bruzzi P., Merlo F., Torcoli R., Nicolo G. (1991). Univariate and multivariate analyses of the relationship between adenocarcinoma and solitary and multiple adenomas in colorectal adenoma patients.. Int J Cancer.

[OCR_01412] Giovannucci E., Stampfer M. J., Colditz G., Rimm E. B., Willett W. C. (1992). Relationship of diet to risk of colorectal adenoma in men.. J Natl Cancer Inst.

[OCR_01417] Hardcastle J. D., Thomas W. M., Chamberlain J., Pye G., Sheffield J., James P. D., Balfour T. W., Amar S. S., Armitage N. C., Moss S. M. (1989). Randomised, controlled trial of faecal occult blood screening for colorectal cancer. Results for first 107,349 subjects.. Lancet.

[OCR_01424] Hoff G., Moen I. E., Trygg K., Frølich W., Sauar J., Vatn M., Gjone E., Larsen S. (1986). Epidemiology of polyps in the rectum and sigmoid colon. Evaluation of nutritional factors.. Scand J Gastroenterol.

[OCR_01434] Hsieh C. C., Maisonneuve P., Boyle P., Macfarlane G. J., Roberston C. (1991). Analysis of quantitative data by quantiles in epidemiologic studies: classification according to cases, noncases, or all subjects?. Epidemiology.

[OCR_01440] Jackson N., Little J., Wilson A. D. (1990). Comparison of diet history interview and self completed questionnaire in assessment of diet in an elderly population.. J Epidemiol Community Health.

[OCR_01448] Kato I., Tominaga S., Matsuura A., Yoshii Y., Shirai M., Kobayashi S. (1990). A comparative case-control study of colorectal cancer and adenoma.. Jpn J Cancer Res.

[OCR_01452] Kune G. A., Kune S., Read A., MacGowan K., Penfold C., Watson L. F. (1991). Colorectal polyps, diet, alcohol, and family history of colorectal cancer: a case-control study.. Nutr Cancer.

[OCR_01464] Macquart-Moulin G., Riboli E., Cornée J., Kaaks R., Berthezène P. (1987). Colorectal polyps and diet: a case-control study in Marseilles.. Int J Cancer.

[OCR_01475] Neugut A. I., Johnsen C. M., Forde K. A., Treat M. R., Nims C. (1988). Vitamin supplements among women with adenomatous polyps and cancer of the colon. Preliminary findings.. Dis Colon Rectum.

[OCR_01485] Newmark H. L., Wargovich M. J., Bruce W. R. (1984). Colon cancer and dietary fat, phosphate, and calcium: a hypothesis.. J Natl Cancer Inst.

[OCR_01492] O'Brien M. J., Winawer S. J., Zauber A. G., Gottlieb L. S., Sternberg S. S., Diaz B., Dickersin G. R., Ewing S., Geller S., Kasimian D. (1990). The National Polyp Study. Patient and polyp characteristics associated with high-grade dysplasia in colorectal adenomas.. Gastroenterology.

[OCR_01507] Stemmermann G. N., Heilbrun L. K., Nomura A. M. (1988). Association of diet and other factors with adenomatous polyps of the large bowel: a prospective autopsy study.. Am J Clin Nutr.

[OCR_01513] Thomas W. M., Hardcastle J. D. (1990). Role of upper gastrointestinal investigations in a screening study for colorectal neoplasia.. Gut.

[OCR_01526] Willett W., Stampfer M. J. (1986). Total energy intake: implications for epidemiologic analyses.. Am J Epidemiol.

[OCR_01518] Willett W. (1989). The search for the causes of breast and colon cancer.. Nature.

[OCR_01531] Wynder E. L., Shigematsu T. (1967). Environmental factors of cancer of the colon and rectum.. Cancer.

[OCR_01535] Zaridze D. G. (1983). Environmental etiology of large-bowel cancer.. J Natl Cancer Inst.

